# Idiopathic Choroidal Neovascularization: Intraocular Inflammatory Cytokines and the Effect of Intravitreal Ranibizumab Treatment

**DOI:** 10.1038/srep31880

**Published:** 2016-08-25

**Authors:** Houfa Yin, Xiaoyun Fang, Jian Ma, Min Chen, Yabo Yang, Shenchao Guo, Zhiqing Chen, Zhaoan Su, Lei Feng, Panpan Ye, Fang Wu, Jinfu Yin

**Affiliations:** 1 Eye Center, Second Affiliated Hospital of Zhejiang University School of Medicine, Hangzhou, China

## Abstract

Idiopathic choroidal neovascularization (ICNV) is a disorder that primarily affecting patients younger than 50 years and can cause severe loss of vision. Choroidal abnormalities, especially choroidal inflammation, have been thought to be involved in the pathophysiology of ICNV. However, the exact pathogenesis of ICNV remains unclear. The aim of our study was investigate the levels of 27 inflammatory cytokines in the aqueous humor of eyes with ICNV, and to determine the effect of intravitreal injection of ranibizumab (IVR) on cytokine levels. Significantly higher levels of IL-2, IL-10, IL-15, IL-17, basic FGF, and GM-CSF were observed in patients with ICNV compared with controls. However, only IL-17 levels were significantly higher in patients with ICNV compared with controls after adjusting for axial length. Furthermore, there were significant correlations between the levels of IL-10, IL-17, GM-CSF, and VEGF and the lesion area. Significant changes in visual acuity and central retinal thickness were observed after IVR. Besides VEGF, IVR also significantly reduced the levels of IL-2, IL-10, basic FGF, and IL-12, however, the IL-6 levels were significantly increased. Our results suggest that there may be an involvement of IL-17-related inflammatory processes in the etiology of ICNV.

Choroidal neovascularization (CNV), which often causes severe vision loss and eventually blindness, is a common pathologic change that may occur in more than 30 ocular diseases^1^. Age-related macular degeneration (AMD) is the most common cause of CNV in the elderly^2^. In patients aged 50 years or younger, CNV may also develop secondary to some underlying conditions such as pathologic myopia (PM), angioid streak, trauma and other inflammatory or hereditary disorders^3^. However, in some young patients with CNV, no apparent cause can be detected, and the CNV is generally categorized as idiopathic CNV (ICNV)^4^.

Choroidal abnormalities, such as focal choroiditis, compensation of choroidal vessels, choroidal ischemia, myopia, or impaired functional activities of circulating hematopoietic stem cells have been thought to be involved in the pathophysiology of ICNV; however, the exact pathogenesis of ICNV is not yet fully understood^3^,^5^,^6^,^7^.

Although the natural progression and the final visual outcomes of ICNV are generally considered to be more favorable than CNV attributable to AMD or PM, severe and irreversible visual loss can occur in some untreated eyes^4^. Nowadays, various ICNV treatments have been reported, such as intravitreal anti-vascular endothelial growth factor (VEGF) therapy or photodynamic therapy (PDT) with verteporfin^8^,^9^,^10^. However, the optimal treatment for ICNV is not well established.

In order to improve ICNV treatment results, the complex molecular mechanisms involved in the pathogenesis of ICNV must be understood more completely. Although inflammatory cytokines such as VEGF and immunoglobulin E were found to be significantly elevated in the serum of patients with ICNV, studies of intraocular inflammatory cytokines in patients with ICNV are sparse^11^. Additionally, little is known about the biologic response of the intraocular milieu to anti-VEGF therapy. Levels of cytokines in the aqueous humor are supposed to reflect their levels in the vitreous, and have been shown to correlate with the corresponding vitreous levels; however, no correlation between cytokine levels in the aqueous humor and plasma or serum has been detected^12^,^13^. Moreover, aqueous humor samples are more easily and safely obtained than vitreous samples.

The purpose of this study was to obtain the profiles of inflammatory cytokines in the aqueous humor of patients with ICNV, and to investigate changes in the levels of various cytokines during anti-VEGF therapy. Furthermore, we also evaluated correlations between cytokines and clinical presentations.

## Results

In this prospective study, 39 eyes of 39 patients with ICNV were included and compared with 28 congenital or age-related cataract control eyes. The clinical characteristics of the 2 groups are summarized in Table 1. There were no significant differences in sex and mean age between the two groups. However, mean axial lengths of the ICNV group were significantly longer than those of the control group. Of the 28 control eyes, 15 eyes were congenital cataract, 13 eyes were age-related cataract. All of the ICNV eyes were phakic. The location of ICNV was subfoveal in 19 eyes (48.72%), juxtafoveal in 10 eyes (25.64%), and extrafoveal in 10 eyes (25.64%). Mean spherical equivalent refractive error was −2.41 ± 1.88 diopters. At baseline, mean best corrected visual acuity (BCVA) was 0.67 ± 0.45 logarithm of the minimum angle of resolution (logMAR), mean central retinal thickness (CRT) was 354.05 ± 86.52 μm in the ICNV patients, and mean lesion area was 2.45 ± 1.75 mm^2^ in the ICNV patients. The mean duration before intravitreal injection of ranibizumab (IVR) in the ICNV group was 56.6 ± 56.2 days (median 30 days, range 7–180 days).

### Cytokine Levels in Aqueous Humor at Baseline Presentation

The levels of 27 types of aqueous humor cytokines in the ICNV and control groups at baseline are listed in Table 2. Compared to the control group, the ICNV group showed significantly higher levels of IL-2, IL-10, IL-15, IL-17, basic FGF, and GM-CSF. However, VEGF levels were not significantly different between the ICNV and control groups. Logistic regression analysis demonstrated that only IL-17 levels were significantly higher in the ICNV group compared with the control group after adjusting for axial length (Table 3).

### Correlations between Aqueous Cytokines and Clinical Parameters

In the ICNV group, no significant correlation was noted between baseline visual acuity (VA) and CRT (*r* = 0.061, *P* = 0.712). The aqueous humor levels of IL-2, IL-10, IL-15, IL-17, basic FGF, GM-CSF, and VEGF did not correlate significantly with baseline VA or CRT (Table 4). However, there were significant correlations between the levels of IL-10, IL-17, GM-CSF, and VEGF and the lesion area (Table 4).

### Changes in Aqueous Cytokines and Clinical Parameters during Treatment

All patients received an initial IVR at their baseline visit. Twenty-four of the 39 patients (61.5%) were retreated according to protocol criteria during a 12-month follow-up period, of which 11, 8, and 5 patients required 2, 3, and 4 injections, respectively. In total, the mean number of ranibizumab injections was 2.1 ± 1.1 during the 12-month follow-up period. We obtained a second aqueous sample from 11 patients who received the second injection 1 month after the first injection.

**Figure 1:**
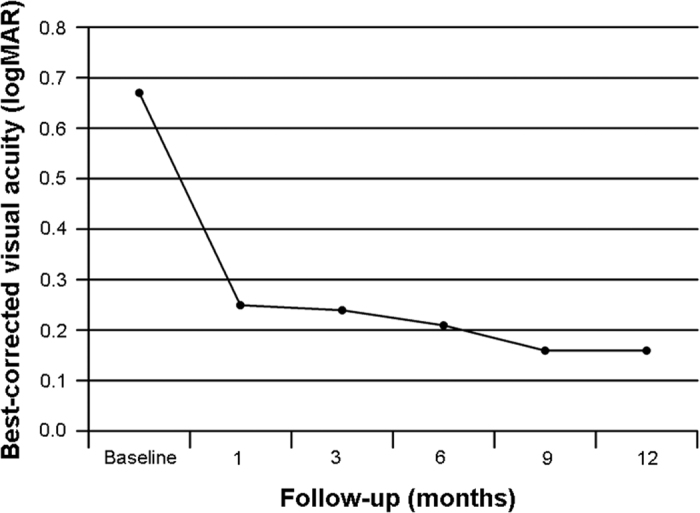
Changes of mean best-corrected visual acuity (BCVA) during 12 months after IVR for ICNV.

**Figure 2:**
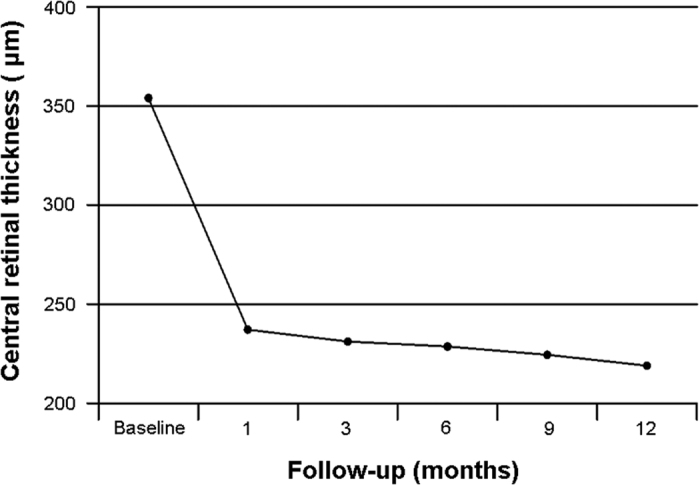
Changes of mean central retinal thickness during 12 months after IVR for ICNV.

The mean BCVA was 0.25 ± 0.28 logMAR and 0.16 ± 0.21 logMAR at 1 month and 12 months, respectively (Fig. 1). There was a statistically significant difference in BCVA at month 1 (*P* < 0.001) and at month 12 (*P* < 0.001) compared with baseline. CRT as measured by OCT demonstrated a statistically significant difference at baseline versus month 1 (*P* < 0.001) and month 12 (*P* < 0.001). The mean CRT was 237.26 ± 30.03 μm and 219.15 ± 19.11 μm at 1 month and 12 months, respectively (Fig. 2). One month after IVR, in the 11 patients, the levels of IL-2, IL-10, and basic FGF were significantly decreased. Furthermore, the VEGF level was significantly decreased from a median of 40.3 pg/ml to 5.7 pg/ml. Moreover, the IL-12 level was significantly decreased. In contrast, the IL-6 levels were significantly increased. Levels of IL-15, IL-17, GM-CSF, and other cytokines were not significantly altered by IVR in the 11 patients (Table 5).

Although our findings showed that IVR therapy was effective for CNV and retinal edema, 24 of the 39 eyes (61.5%) needed reinjections (refractory group). We sought to identify the exacerbating cytokine or cytokines by comparing refractory group (subgroup B) with improved group (subgroup A). There was no significant difference between the two groups in terms of sex, age, axial length, duration before IVR, spherical equivalent, CNV location, baseline BCVA, CRT, or lesion area (see Supplementary Table S1). When the baseline cytokine levels of both groups were compared, we found that the levels of IL-10 and MIP-1b were significantly higher in subgroup B than in subgroup A (Table 5).

## Discussion

Using the multiplex bead immunoassays, we found significantly higher levels of IL-17 in the aqueous humor of ICNV patients. To the best of our knowledge, our study was the first to analyze the distribution of various inflammatory cytokines in the aqueous humor of ICNV patients and to assess their changes after IVR treatment.

It is now quite evident that the development and progression of CNV is associated with alterations in various pro- and anti-angiogenic factors^14^. The collective evidence suggests that VEGF is a critical angiogenic factor for promoting ocular angiogenesis^15^. Ocular anti-VEGF therapy is highly effective for treating CNV, however, it is important to note that not all patients respond to such therapy, which suggests that VEGF-driven pathways are only part of the complex processes regulating angiogenesis, and that other molecules besides VEGF may play a crucial role in aberrant angiogenesis^15^. Increasing evidence suggests that inflammation and the immune system may be involved in the development of CNV^15^,^16^,^17^.

Interestingly, we found significantly elevated aqueous humor levels of IL-17 in the ICNV eyes. IL-17, the signature cytokine of T-helper 17 (Th17) cells, is an inflammatory cytokine that plays a crucial role in the pathogenesis of autoimmune and inflammatory diseases, including rheumatoid arthritis, psoriasis, uveitis, and scleritis, by inducing the expression of inflammatory cytokines and chemokines^18^,^19^. Additionally, IL-17 also promotes the angiogenesis effect of VEGF, basic FGF, and hepatocyte growth factor^20^. Compelling evidence suggests that IL-17 could be involved in the pathogenesis of neovascular AMD (nAMD) by promoting CNV^21^. Elevated serum levels of IL-17 and other inflammatory cytokines were recently shown in nAMD patients^21^. Moreover, Wei *et al*. have demonstrated the elevated expression of IL-17A and its receptor IL-17RC in the macular tissues of nAMD patients^22^. Importantly, Hasegawa *et al*. have demonstrated that IL-17 has a strong potential for stimulating neovascularization in a VEGF-independent manner in laser-induced experimental CNV model^23^. Furthermore, the suppressive effect of anti-IL-17 therapy on CNV volume and area was similar to that of anti-VEGF therapy, and the combination of anti-VEGF and anti-IL-17 therapy was more effective^23^. The observed presence of elevated intraocular IL-17 in our study indicates that IL-17 may be involved in the pathogenesis of ICNV. Furthermore, IL-17 was not remarkably inhibited by the preceding anti-VEGF therapy. Considering the effect of anti-IL-17 treatment in laser-induced experimental CNV, we speculate that IL-17 could potentially be an additional target molecule in therapy for ICNV. Indeed, anti-IL-17 therapy inhibits multiple inflammatory cytokines and appears to be effective for both rheumatoid arthritis and psoriasis in clinical trials^24^,^25^.

In our study, the aqueous VEGF levels measured in the eyes of control patients with cataract were aligned with study by Funk *et al*., in which multiplex bead immunoassays were also used^26^. However, no significant difference in the VEGF levels was noted between the ICNV group and the control group. This finding was well supported by various previous studies that VEGF levels in the aqueous humor of patients with nAMD were not significantly different from patients with cataract although there were some contradictory results^26^,^27^,^28^,^29^,^30^,^31^. It was also interesting to note that anti-VEGF treatment significantly decreased VEGF levels although VEGF levels were not necessarily elevated in aqueous humour, which was consistent with the results of the previous studies^28^,^30^. The total levels of cytokines in the ocular fluid may be affected by the area of CNV lesions^30^,^32^. Correspondingly, the aqueous humor levels of IL-10, IL-17, GM-CSF, and VEGF were positively correlated with the lesion area in our study. Because the relatively small lesion area, we speculate that the levels of some inflammatory cytokines, including VEGF, may not be high enough to show statistical difference from the control in the aqueous humor although they are elevated in the lesion site and vitreous fluid in ICNV^29^,^30^.

Our study demonstrated that inflammatory cytokine levels other than VEGF, including IL-2, IL-10, IL-12, and basic FGF were also decreased after IVR in the refractory group. Although aqueous humor levels of IL-2, IL-10, and basic FGF were not significantly different between the ICNV group and the control group after adjusting for axial length, these cytokines appear to be involved in the mechanism of CNV as well. IL-2, a T-helper, lymphocyte-derived cytokine, upregulates VEGF expression *in vitro*^33^,^34^. Although the precise mechanism through which IL-10 promotes angiogenesis in the eye remains unclear, recent studies have demonstrated that it promotes angiogenesis either by preventing the infiltration of anti-angiogenic macrophages into the choroid or by directly polarizing macrophages towards a proangiogenic phenotype^35^,^36^. Basic FGF is a potent angiogenic molecule that occupied the central stage in the angiogenesis field before the discovery of the VEGF family^37^. Previous studies have demonstrated that basic FGF is involved in the pathogenesis of proliferative diabetic retinopathy^38^. However, aqueous IL-6 concentration was significantly increased after IVR. IL-6 is an important inflammatory cytokine, which are increased in patients with ischemic retinal disorders^39^,^40^. It induces numerous angiogenic and inflammatory cytokines directly or indirectly, including VEGF, which may explain the suboptimal response to IVR in the refractory group of our study^41^. However, further investigations are necessary to determine the exact underlying effect of anti-VEGF treatment in other intraocular inflammatory cytokines.

Previous studies have reported promising results with anti-VEGF therapy in ICNV patients^8^,^9^,^10^,^42^. Zhang *et al*. reported that the mean number of bevacizumab injections was 2 during the 12-month follow-up, and 40.0% and 70.0% of eyes had complete resolution of fluid after a single or an additional injection, respectively^9^. Similarly, the mean number of ranibizumab injections was 2.1 during the 12-month follow-up in our study. It suggests that the number of injections does not differ significantly between ranibizumab and bevacizumab in treatment of ICNV. Another important result of this study was the significantly elevated aqueous humor levels of IL-10 and MIP-1b in the refractory group of our study. These cytokines may play a role in the inflammation-induced angiogenesis by regulating macrophages^35^,^36^,^43^. Some more studies are needed to allow a better understanding of the pathogenesis of ICNV.

The present study has several limitations. First, we were unable to measure the cytokine levels in the aqueous humor of all the ICNV patients 1 month later after the first injection. Since we collected aqueous humor samples just before IVR, patients without recurrent macular edema did not receive IVR. Second, although our study identified key inflammatory cytokines in ICNV patients and investigated their changes during anti-VEGF therapy, we did not reveal any potential mechanisms. Further animal experiments and *in vitro* experiments are required to determine the mechanism by which these cytokines are correlated with ICNV.

In conclusion, this study investigated a profile of inflammatory cytokines in the aqueous humor of patients with ICNV. We found that IL-17 levels were increased significantly in the aqueous humor of patients with ICNV. This suggests that there may be an involvement of IL-17-related inflammatory processes in the etiology of ICNV and that may facilitate new treatments for this vision-threatening disease.

## Methods

### Study Subjects

This study was performed at the Eye Center, Second Affiliated Hospital of Zhejiang University School of Medicine. The study protocol was approved by the Ethics Committee of the Second Affiliated Hospital of Zhejiang University School of Medicine, and the procedures used conformed to the tenets of the Declaration of Helsinki. Informed consent was obtained from all patients before study inclusion. Thirty-nine consecutive ICNV patients who were scheduled to undergo intravitreal injection of 0.5 mg ranibizumab (Lucentis; Novartis Pharma AG, Basel, Switzerland) were studied.

Inclusion criteria for ICNV in this study were: (1) patients younger than 50 years; (2) previous untreated CNV; (3) OCT showing intraretinal edema, subretinal fluid (SRF), or pigment epithelial detachment (PED); and (4) absence of concurrent ocular diseases in the study eyes that compromised or could have compromised vision and ocular condition. Exclusion criteria were: (1) clinical features suggesting that CNV was secondary to other causes such as AMD, PM, trauma, or hereditary diseases; (2) axial length >26.0 mm or myopia> −6 diopter; (3) history of prior treatment for ICNV, including intravitreal drug injection, PDT, systemic or focal steroids; (4) previous intraocular surgery.

### Diagnostic Procedures and Follow-up

All eyes underwent a full ophthalmological examination at baseline, including BCVA testing using a Snellen chart, slit-lamp biomicroscopy, dilated fundus examination, intraocular pressure (IOP) measurement, color fundus photography, axial length (IOLMaster; Carl Zeiss Meditec, Dublin, CA, USA), fundus fluorescein angiography (FFA, Heidelberg Engineering HRA Spectralis, Heidelberg, Germany), and OCT (Cirrus OCT; Carl Zeiss Meditec, Dublin, CA, USA). CRT (thickness of the 1-mm central retina) was measured by the fast macular thickness map scan modes. Lesion area, including dye leakage area, PED, subretinal hemorrhage, and hyperfluorescent staining of fibrous tissue was measured as described previously^44^. For statistical analysis, BCVA were converted to logMAR units. Follow-up examinations were performed on 1 day, 1 week, 1 month, 2 months, 3 months, and every 3 months thereafter over a period of 12 months. However, patients were asked to come earlier in cases of visual loss and/or recurrence of metamorphopsia. Slit-lamp biomicroscopy, dilated fundus examination, IOP measurement, and the measurements of BCVA and CRT were performed at each study visit. Additional FFA and color fundus photography was performed if physicians suspected vision loss had occurred without associated signs of recurrent macular edema by OCT. IVR was repeated if OCT showed persistent intraretinal edema, SRF, or PED. Patients were defined as refractory to IVR treatment if they needed reinjections. Therefore, all patients were divided into 2 subgroups: the improved group (subgroup A) included patients who received 1 injection during the 12-month follow-up period, and the refractory group (subgroup B) included patients who received 2 or more injections during the 12-month follow-up period.

### Intravitreal Injection of Ranibizumab and Sample Collection

All patients received an initial IVR as described previously^45^. Immediately before intravitreal injection, approximately 0.05–0.1 mL of undiluted aqueous humor was collected by performing an anterior chamber limbal paracentesis, and the aqueous samples were stored at −80 °C until processing. We obtained aqueous samples from all patients before the first injection. We also obtained a second aqueous sample from 11 patients who received the second injection 1 month after the first injection.

### Control Group

Control aqueous samples were obtained from 28 eyes of 28 congenital or age-related cataract patients who underwent cataract surgery by limbal paracentesis, and the samples were stored at −80 °C until processing. Exclusion criteria were the following: (1) any type of retinal disease, uveitis, or glaucoma; (2) previous intraocular surgery; (3) diabetes mellitus, use of immunosuppressive drugs, or malignant tumors any location; and (4) axial length >26.0.

### Multiplex Bead Analyses

A Bio-Plex multiplex assay (Bio-Plex Human Cytokine 27-plex panel; Bio-Rad, Hercules, CA, USA) and a multiplex bead analysis system (Bio-Plex Suspension Array System; Bio-Rad) were used simultaneously to measure the levels of 27 types of cytokines in the aqueous humor, as previously described^46^,^47^,^48^. The following were analyzed: IL-1b, IL-1ra, IL-2, IL-4, IL-5, IL-6, IL-7, IL-8, IL-9, IL-10, IL-12, IL-13, IL-15, IL-17, Eotaxin, basic FGF, G-CSF, GM-CSF, IFN-γ, IP-10, MCP-1, MIP-1a, MIP-1b, PDGF-bb, RANTES, TNF-α, and VEGF. The levels of aqueous humor cytokines were set to 0 if the levels were below the detectable levels.

### Statistical Analysis

Statistical analyses were performed using SPSS software version 17.0 (SPSS, Inc., Chicago, IL, USA). Data were recorded as the means ± standard deviation (SD) or the median and range. To evaluate the differences between groups, unpaired *t* test or Mann-Whitney *U* test was used to determine their significance. The χ^2^ test or Fisher exact test was used to compare categorical variables. Paired *t* test or Wilcoxon signed rank test was used to compare within-group categorical variable changes from baseline. Spearman’s correlation analysis was used to evaluate the relationship between numerical data. Logistic regression analysis was carried out to confirm the association of elevated cytokines with ICNV. *P* < 0.05 was considered to be statistically significant.

## Additional Information

**How to cite this article**: Yin, H. *et al*. Idiopathic Choroidal Neovascularization: Intraocular Inflammatory Cytokines and the Effect of Intravitreal Ranibizumab Treatment. *Sci. Rep.* **6**, 31880; doi: 10.1038/srep31880 (2016).

## Supplementary Material

Supplementary Information

## Figures and Tables

**Table 1:** Clinical characteristics of the patients. Data are presented as mean ± SD or n (%).

Characteristic	ICNV Group (n = 39)	Control Group (n = 28)	*P* value
Mean age of patients, yrs	35.1 ± 7.8	35.6 ± 12.1	0.533
Sex, n (%)			0.400
Male	15 (38.5)	8 (28.6)	
Female	24 (61.5)	20 (71.4)	
Mean axial length, mm	24.18 ± 1.15	23.06 ± 1.08	<0.001

**Table 2:** Aqueous humor levels of 27 types of cytokines in the ICNV and control groups.

	ICNV Group (n = 39)	Control Group (n = 28)	*P* Value
**IL-2**	**1.8 (0–11.2)**		**<0.001**
**IL-10**	**4.3 (2.2–15.2)**	**3.6 (0–16.1)**	**0.039**
**IL-15**	**7.6 (0–27.8)**	**5.9 (0–39.6)**	**0.049**
**IL-17**	**9.5 (0–55.7)**	**0 (0–45.7)**	**<0.001**
**basic FGF**	**13.2 (5.0–88.4)**	**7.2 (0–79.6)**	**<0.001**
**GM-CSF**	**169.0 (35.6–1450.1)**	**131.0 (0–1533.1)**	**0.001**
IL-1b	0 (0–3.2)	0	0.397
IL-1ra	0 (0–131.3)	0 (0–26.1)	0.847
IL-4	0	0 (0–0.4)	0.238
IL-5	0	0	—
IL-6	3.2 (0–120.9)	5.1 (0–15.6)	0.346
IL-7	1.9 (0–3.6)	2.0 (0–11.4)	0.418
IL-8	0 (0–4.5)	0 (0–6.9)	0.668
IL-9	0 (0–5.2)	0 (0–3.1)	0.157
IL-12	10.3 (0–25.0)	9.5 (0–29.9)	0.559
IL-13	2.0 (0–6.5)	1.6 (0–8.4)	0.220
Eotaxin	0	0 (0–2.3)	0.238
G-CSF	0	0	—
IFN-γ	0 (0–9.6)	0	0.397
IP-10	47.7 (0–407.4)	69.5 (0–184.9)	0.162
MCP-1	338.9 (180.7–596.7)	334.8 (15.9–544.6)	0.575
MIP-1a	0.7 (0–3.9)	0 (0–4.1)	0.152
MIP-1b	7.1 (1.9–22.0)	8.2 (1.1–19.8)	0.100
PDGF-bb	0	0 (0–1.4)	0.238
RANTES	0	0 (0–25.8)	0.093
TNF-α	0	0	—
VEGF	40.9 (17.6–108.8)	38.9 (0–105.9)	0.869

Values in boldface type indicate statistical significance. Levels are expressed as the median (range) pg/ml.

**Table 3:** Association of ICNV with cytokines in aqueous humor by logistic regression analysis.

	OR	95% CI	*P* Value
IL-2	1.259	(0.967–1.638)	0.087
IL-10	1.252	(1.000–1.569)	0.052
IL-15	1.046	(0.958–1.141)	0.361
IL-17	1.131	(1.019–1.256)	0.021
basic FGF	1.068	(0.997–1.144)	0.059
GM-CSF	1.002	(0.999–1.004)	0.183
VEGF	1.010	(0.987–1.034)	0.388

*P* values are adjusted for axial length.

**Table 4:** Correlations between aqueous humor cytokines and visual acuity, central retinal thickness, or lesion area.

Variables	Visual Acuity		Central Retinal Thickness		Lesion Area	
	***r***	***P*** Value	***r***	***P*** Value	***r***	***P*** Value
IL-2	0.272	0.095	−0.147	0.372	0.372	0.051
IL-10	0.282	0.082	−0.118	0.475	0.538	0.003
IL-15	0.165	0.316	−0.120	0.468	0.316	0.101
IL-17	0.304	0.060	−0.280	0.084	0.520	0.005
basic FGF	0.108	0.514	−0.287	0.077	0.286	0.140
GM-CSF	0.293	0.070	−0.255	0.117	0.454	0.015
VEGF	0.164	0.318	0.050	0.763	0.465	0.013

Correlation coefficient (*r*) and *P* values are calculated by Spearman’s correlation.

**Table 5:** Aqueous humor levels of 27 types of cytokines in subgroup A and subgroup B and changes after IVR.

	Subgroup A (n = 15)	Subgroup B (n = 24)	Preinjection (n = 11)	Postinjection (n = 11)	*P* Value	
							Subgroup A vs Subgroup B	Preinjection vs Postinjection
IL-2	1.3 (0–3.3)	1.8 (0–11.2)	2.0 (0–11.2)	1.8 (0–2.8)	0.187	0.028
IL-10	4.1 (2.8–6.1)	5.0 (2.2–15.2)	5.3 (2.2–15.2)	2.4 (0–4.3)	0.043	0.004
IL-15	6.6 (3.5–10.5)	7.9 (0–27.8)	8.1 (3.8–18.1)	7.8 (3.1–10.3)	0.112	0.175
IL-17	9.5 (0–15.1)	9.8 (0–55.7)	9.5 (0–55.7)	9.2 (0–11.2)	0.675	0.221
basic FGF	13.4 (7.5–28.3)	12.9 (5.0–88.4)	12.5 (5.0–88.4)	10. 0 (0–42.4)	0.544	0.041
GM-CSF	169.0 (114.2–291.8)	171.8 (35.6–1450.1)	207.6 (71.5–1450.1)	165.4 (39.6–259.9)	0.624	0.075
IL-1b	0 (0–3.2)	0	0	0	0.206	—
IL-1ra	0 (0–12.0)	5.0 (0–131.3)	0 (0–131.3)	0 (0–29.4)	0.122	0.612
IL-4	0	0	0	0 (0–0.3)	—	0.317
IL-5	0	0	0	0	—	—
IL-6	3.4 (0–7.5)	3.0 (0–120.9)	2.7 (0–120.9)	4.5 (1.9–698.2)	0.897	0.016
IL-7	1.6 (0–3.2)	2.0 (0–3.6)	2.1 (0–3.6)	1.5 (0–3.4)	0.056	0.663
IL-8	0	0 (0–4.5)	0 (0–4.1)	0 (0–16.5)	0.100	0.317
IL-9	0 (0–2.0)	0 (0–5.2)	0 (0–1.4)	0 (0–1.4)	1.000	1.000
IL-12	10.0 (4.2–15.3)	11.5 (0–25.0)	10.3 (0–25.0)	0 (0–9.1)	0.238	0.008
IL-13	1.8 (0.6–6.5)	2.0 (0–5.4)	2.1 (0–5.4)	1.7 (0.5–3.4)	0.462	0.386
Eotaxin	0	0	0	0	—	—
G-CSF	0	0	0	0 (0–12.8)	—	0.317
IFN-γ	0	0 (0–9.6)	0	0 (0–9.3)	0.429	0.317
IP-10	59.4 (0–202.7)	39.4 (0–407.4)	32.2 (0–407.4)	46.2 (1.6–1375.8)	0.231	0.110
MCP-1	331.5 (180.7–443.7)	346.4 (185.5–596.7)	343.2 (206.1–476.8)	311.2 (171.6–625.9)	0.335	0.648
MIP-1a	0.7 (0–1.1)	0 (0.3–3.9)	0 (0–3.9)	0.7 (0–0.8)	0.616	0.674
MIP-1b	5.1 (1.9–13.3)	7.9 (3.2–22.0)	7.1 (3.2–13.5)	7.0 (4.0–15.4)	0.030	0.407
PDGF-bb	0	0	0	0	—	—
RANTES	0	0	0	0	—	—
TNF-α	0	0	0	0 (0–5.4)	—	0.317
VEGF	37.5(18.5–69.1)	44.8(17.6–108.8)	40.3 (17.6–108.8)	5.7 (0–59.9)	0.097	0.003

Values in boldface type indicate statistical significance. Levels are expressed as the median (range) pg/ml.
