# Supplementary material for: Idiopathic Choroidal Neovascularization: Intraocular Inflammatory Cytokines and the Effect of Intravitreal Ranibizumab Treatment
Source: Sci Rep. 2016 Aug 25;6:31880. doi: 10.1038/srep31880 (PMC4997256; doi:10.1038/srep31880)
Supplement: Supplementary Information [file srep31880-s1.pdf]

Idiopathic Choroidal Neovascularization: Intraocular Inflammatory Cytokines and the Effect of Intravitreal Ranibizumab Treatment

Houfa Yin, Xiaoyun Fang*, Jian Ma, Min Chen, Yabo Yang, Shenchao Guo, Zhiqing Chen, Zhaoan Su, Lei Feng, Panpan Ye, Fang Wu & Jinfu Yin

Eye Center, Second Affiliated Hospital of Zhejiang University School of Medicine, Hangzhou, China. Correspondence and requests for materials should be addressed to X.F.(email: xiaoyunfang@zju.edu.cn)

	Subgroup A (n=15)	Subgroup B (n=24)	*P* value
Sex, n (%)			0.317
Male	4 (26.7)	11 (45.8)	
Female	11 (73.3)	13 (54.2)	
Mean age of patients, yrs	32.1 ±7.4	36.9 ±7.7	0.062
Mean axial length, mm	24.34 ±1.14	24.09 ±1.18	0.512
Mean duration before IVR, d	52.6 ±59.6	59.2 ±55.2	0.442
Mean spherical equivalent, D	2.93 ±1.83	2.10 ±1.87	0.144
Location of CNV			0.807
(subfoveal/juxtafoveal/extrafoveal), n	8/4/3	11/6/7	
BCVA, logMAR			
Baseline	0.75 ±0.53	0.63 ±0.39	0.582
Month 1	0.23 ±0.31	0.27 ±0.26	0.332
Month 12	0.17 ±0.26	0.15 ±0.17	0.820
CRT, um			
Baseline	335.87 ±93.89	365.42 ±81.56	0.306
Month 1	233.87 ±26.95	239.38 ±32.19	0.795
Month 12	215.27 ±19.46	221.58 ±19.90	0.322
Lesion area, mm^2			
Baseline	2.63 ±1.98	2.31 ±1.62	0.646

Supplementary Table S1. Clinical characteristics of subgroup A and subgroup B. Subgroup A, patients who received 1 injection during the 12-month follow-up period. Subgroup B, patients who received 2 or more injections during the 12-month follow-up period. Data are presented as mean ± SD or n (%).
